# The association of metformin use with vitamin B12 deficiency and peripheral neuropathy in Saudi individuals with type 2 diabetes mellitus

**DOI:** 10.1371/journal.pone.0204420

**Published:** 2018-10-15

**Authors:** Turki J. Alharbi, Ayla M. Tourkmani, Osama Abdelhay, Hesham I. Alkhashan, Abdulrahman K. Al-Asmari, Abdulaziz M. Bin Rsheed, Sarah N. Abuhaimed, Najeebuddin Mohammed, Abdulrhman N. AlRasheed, Nouf G. AlHarbi

**Affiliations:** 1 Family and Community Medicine Department, Prince Sultan Medical City, Riyadh, Kingdom of Saudi Arabia; 2 Research Center, Prince Sultan Military Medical City, Riyadh, Kingdom of Saudi Arabia; Weill Cornell Medicine-Qatar, QATAR

## Abstract

**Aims:**

To compare the prevalence of vitamin B12 deficiency and peripheral neuropathy between two groups of type 2 diabetes mellitus (T2DM) patients treated with or without metformin, and to determine factors associated with vitamin B12 deficiency therapy and dietary intake of vitamin B12.

**Methods:**

In this retrospective study, we recruited 412 individuals with T2DM: 319 taking metformin, and 93 non-metformin users. Demographics, dietary assessment for vitamin B12 intakes, and medical history were collected. Participants were assessed for peripheral neuropathy. Blood specimens were collected and checked for serum vitamin B12 levels. The differences between the two groups were analyzed using an independent t-test for continuous data, and the Chi-squared or Fisher's exact test was used for categorical data. The relationship of vitamin B12 deficiency with demographics and clinical characteristics was modeled using logistic regression.

**Results:**

The prevalence of B12 deficiency was 7.8% overall, but 9.4% and 2.2% in metformin users and non-metformin users, respectively. The odds ratio for serum vitamin B12 deficiency in metformin users was 4.72 (95% CI, 1.11–20.15, *P* = 0.036). There were no significant differences in a test of peripheral neuropathy between the metformin users and non-metformin users (*P* > 0.05). Low levels of vitamin B12 occurred when metformin was taken at a dose of more than 2,000 mg/day (AOR, 21.67; 95% CI, 2.87–163.47) or for more than 4 years (AOR, 6.35; 95% CI, 1.47–24.47).

**Conclusion:**

Individuals with T2DM treated with metformin, particularly those who use metformin at large dosages (> 2,000 mg/day) and for a longer duration (> 4 years), should be regularly screened for vitamin B12 deficiency and metformin is associated with B12 deficiency, but this is not associated with peripheral neuropathy.

## Introduction

Metformin is the first therapeutic choice for management of type 2 diabetes mellitus (T2DM), as recommended by the American Diabetes Association and the European Association for the Study of Diabetes. Metformin improves peripheral insulin sensitivity and cardiovascular mortality risk [1, 2]. Moreover, metformin has beneficial effects on carbohydrate metabolism, weight loss, and vascular protection [3]. Most of the side effects associated with metformin are mild, such as abdominal distress and diarrhea [4]. However, metformin is reported to diminish cobalamin (vitamin B12) uptake in the terminal ileum [5]. Several studies have shown that long-term use of metformin leads to malabsorption of vitamin B12, with a decrease in the concentration of serum vitamin B12 from 30% to 14% [6]. Additionally, randomized control trials and cross-sectional studies have reported a decrease in serum vitamin B12 level between 9% and 52% with metformin use [5, 7]. A systematic review by Chapman et al. (2016) of individuals with T2DM found 10 out of 17 observational studies reported that metformin users had significantly lower levels of vitamin B12 than non-metformin users. Furthermore, a meta-analysis within this review of four clinical trials demonstrated that metformin significantly reduced overall vitamin B12 levels after three to six months of use [8].

Ting et al. (2006) reported that the dose and duration of metformin use were directly correlated with reduction in levels of serum vitamin B12 [6]. Furthermore, because vitamin B12 participates in an essential pathway of homocysteine metabolism, a reduction in vitamin B12 would increase the plasma level of homocysteine, which is strongly linked to cardiovascular prognosis in T2DM [9].

Metformin use is associated with vitamin B12 deficiency and can result in neuropathy, ranging from paresthesia and decreased peripheral sensation to changes in mental status [1]. Unfortunately, the symptoms of diabetic neuropathy overlap with impaired vibration sensation and proprioception, as well as paresthesia, which has also been found to be associated with vitamin B12 deficiency [10]. Therefore, it has been suggested that neuropathy and vitamin B12 deficiency symptoms should be routinely assessed in individuals with diabetes mellitus, via standard neurological examination, and graded using the Toronto Clinical Scoring System (TCSS) with detection and scoring of B12 deficiency symptoms [11]. This would inform the need for routine annual vitamin B12 measurements in individuals with T2DM who are using metformin.

There is a lack of studies in Saudi Arabia on the prevalence of metformin-related vitamin B12 deficiency in individuals with T2DM. Additionally, there are no guidelines to address how often individuals with T2DM who are being treated with metformin should be screened for vitamin B12 deficiency risk and, if appropriate, prescribed vitamin B12 supplements.

## Materials and methods

### Study design, setting, and recruitment

This study deployed an observational retrospective design. All eligible participants with T2DM were recruited between January and June of 2016 from a primary care center managed by the Prince Sultan Military Medical City (PSMMC) located in Riyadh, Saudi Arabia. A list of potential participants was developed by the researchers based on data obtained from their medical records. Then, during their regular clinical visit, potential participants were assessed by the researchers and were asked for their willingness to participate in the study. Before enrollment, if the potential participants accepted the invitation to be part of this study, they were asked to sign a consent form compatible with the Helsinki Declaration of the World Medical Association. This study was part of a larger study approved by the Institutional Review Board at the PSMMC.

Participants were eligible to be part of the study if they were diagnosed with T2DM and were above 18 years of age. Participants were excluded from the study if they had a history of pernicious anemia, chronic renal failure, cancer, Addison's diseases, intestinal tuberculosis, celiac disease, irritable bowel diseases, or gastric iliac surgery. Also, participants were excluded if they had been taking metformin for less than one year or were taking any of the following medications: phenytoin, primidone, colchicine, isoniazid, hydralazine, or methotrexate. Also, the exclusion criteria were extended to include pregnant and vegetarian participants.

### Variables and data collection

The following data were obtained from the participants: age, gender, household income, intake of vitamin B12 supplement, and a dietary assessment for vitamin B12 intake using information derived from a validated and reliable ADA survey. Participants’ medical records were used to obtain the following data: (1) diabetes-related information; (2) medication history; and (3) past medical history.

The following data were also obtained: (1) physical measures: weight, height, BMI, systolic and diastolic blood pressure; and (2) neurological examination for neuropathy using the Toronto Clinical Neuropathy Score (TCSS), which is a validated and reliable scale for the diagnosis and staging of diabetic sensorimotor polyneuropathy. TCSS was assessed by presence or absence of general symptoms, including foot pain, numbness, tingling, weakness, ataxia, and upper limb symptoms. Based on TCSS score, patients were graded into four classes: no neuropathy, 0–5; mild neuropathy, 6–9; moderate neuropathy, 10–12; and severe neuropathy, >12, with a maximum score of 19. In addition to the TCSS for neuropathy, vitamin B12 deficiency symptoms were also assessed (sore tongue and shortness of breath), and the patients underwent a sensory examination that included pinprick, temperature, light touch, vibration, and position sense. On the basis of the sensory examination, patients were categorized as normal or abnormal in both legs. The Toronto Scoring for DSP included reflexes (knee reflexes, ankle reflexes, and the plantar reflex), and further extended examination was conducted to assess power, tone, gait, and Romberg's sign.

The Focused Neuropathy Test was used to evaluate the presence and severity of diabetic peripheral sensorimotor polyneuropathy. Based on their TCSS scores, participants were graded into four categories of neuropathy: no neuropathy, 0–5; mild neuropathy, 6–9; moderate neuropathy, 10–12; and severe neuropathy for scores > 12. Total TCSS scores ranged from 0 to 19. The Focused Neuropathy Test was used to evaluate specific symptoms associated with vitamin B12 deficiency, and on this basis, participants were categorized into positive or negative status.

A blood specimen was obtained from each participant and the following analyses were carried out: level of serum creatinine, albumin/creatinine ratio (ACR), thyroid function test (TFT), hemoglobin (Hgb), mean corpuscular volume (MCV), homocysteine, folate, vitamin D, and vitamin B12. Serum vitamin B12 was determined via electrochemiluminescence immune assay (ECLIA) (Roche Elecsys 2010 analyzer; Switzerland). Vitamin B12 deficiency was categorized into three groups: mild (118.1–132.8 pmol/L), moderate (88.6–118.1 pmol/L), and severe (< 88.56 pmol/L) deficiency. Homocysteine level was also measured in our study, as an indicator for vitamin B12 deficiency. The normal reading for serum homocysteine should be less than 10 μmol/L, so any specimen with a homocysteine level above this cutoff was considered elevated, and this elevation was interpreted as another indicator of vitamin B12 deficiency.

### Sample size estimation and statistical analysis

STATA 13 was used in our estimation of the minimal sample size needed for valid results, and in our statistical analysis. Minimum sample size was calculated to ensure sufficient power to analyze our primary outcome (vitamin B12 deficiency), and was calculated based on the following parameters: two-sided t test, alpha = 0.05, power = 0.80, with an assumed incidence of vitamin B12 deficiency of 25% in the metformin user group and 5% in the non-metformin user group, based on previous studies. On this basis, the minimum total sample size was calculated as 98. Our study enrolled 412 participants, which the above calculation indicates as providing adequate power.

All nominal and ordinal data were reported in frequencies and percentages, and numerical data were reported in means, and standard deviations. The differences between the two groups were analyzed using an independent t-test for continuous data, and the Chi-squared or Fisher's exact test was used for categorical data. The relationship of vitamin B12 deficiency with demographics and clinical characteristics was modeled using logistic regression. This modeling was conducted in two stages: (1) dependent variable with each independent variable considered individually, which produced unadjusted odds ratios (OR); and (2) a second stage which adjusted for covariates and produced the adjusted odds ratios (AOR). Receiver operator characteristic (ROC) curve analysis was used to determine the model-predictive level with an area under the curve (AUC). The AUC with a 95% confidence interval (CI) was calculated. All hypotheses were tested as two-sided, with a significance level of p ≤ 0.05 and 95% confidence intervals.

## Results

This study enrolled 412 participants with T2DM, who were divided into two groups: those who were taking metformin (metformin users) and those who were not taking metformin (metformin non-users) (see Table 1).

**Table 1:** Participants’ demographic and clinical characteristics, segregated on the basis of metformin use (N = 412).

Characteristics	Group		*P*-value*
	Non-metformin user (n = 93) Mean (SD) or n (%)	Metformin user (n = 319) Mean (SD) or n (%)	
**Gender**			
**Male**	49 (52.69)	147 (46.10)	0.26
**Female**	44 (74.31)	172 (53.90)	
**Age (years)**	56.6 ± 1.4	57.8 ± 0.6	0.35
**Income (SAR)**†			
**< 3,000**	22 (23.66)	85 (26.65)	0.75
**3,000–6,000**	29 (31.18)	85 (26.65)	
**6,000–9,000**	13 (13.98)	54 (16.93)	
**> 9,000**	29 (31.18)	95 (29.77)	
**B12 intake (mcg/week)**	23.0 ± 0.8	23.7 ± 0.5	0.51
**Below RMDI**‡	22 (23.66)	70 (21.94)	0.78
**Above or equal RMDI**	71 (76.34)	249 (78.06)	
**Folate level (nmol/L)**	26.3 ± 8.0	23.3 ± 9.6	<0.01
**Vitamin B12 Level (pmol/L)**	365.37±189.88	313.69±141.84	0.016
**Homocysteine level (μmol/L)**	13.40±5.67	22.165±21.84	<0.001
**Vitamin D** (nmol/L)	46.89±28.28	66.56±24.13	<0.001
**FBS**	10.30±4,71	9.98±5.14	0.589
**HbA**_**1c**_	8.29±1.81	8.05±1.58	0.197
**Weight**	85.1 ± 2.1	83.0 ± 1.0	0.33
**BMI**§	32.6 ± 0.8	32.3 ± 0.5	0.80
**Duration of Diabetes (years)**	7.3 ± 0.8	10.1 ± 0.4	<0.01
**Duration of Metformin Intake (years)**			
**< 1**	n/a	1 (0.31)	n/a
**1–4**	n/a	123 (38.56)	
**> 4**	n/a	196 (61.13)	
**Metformin Dose (mg)**			
**< 1,000**	n/a	6 (1.88)	n/a
**1,000–2,000**	n/a	301 (94.36)	
**> 2,000**	n/a	12 (3.76)	
**Toronto Clinical Scoring System**			
**No Neuropathy**	75 (80.65)	223 (69.91)	0.12
**Mild**	14 (15.05)	71 (22.26)	
**Moderate**	4 (4.30)	25 (7.83)	
**Focused Neurological Test**			
**Negative**	80 (86.02)	279 (87.46)	0.72
**Positive**	13 (13.98)	40 (12.54)	

* Fisher’s exact test and the Chi-squared test of association are used for the comparison between categorical variables, and two-samples independent t-test is used for the comparison between continuous variables.

^†^ SAR: Saudi Riyal, 1 USD = 3.75 SAR

^‡^ RMDI: Recommended Minimum Daily Intake (2.4 mcg/day or 16.8 mcg/week)

^§^ BMI: Body Mass Index

The metformin-using group included 319 participants with an average age of 57.8 ± 0.6 years, and the non-metformin users included 93 participants with an average age of 56.6 ± 1.4 years. Age, sex, and income were similar in both groups, with no significant differences (P = 0.2, 0.3, and 0.7 for sex, age, and income, respectively). The two groups were not significantly different in weight and BMI (P = 0.3 and 0.8, respectively). The dietary intake of vitamin B12 did not differ significantly between the two studied groups (P = 0.7), and the mean supplemental vitamin B12 taken by the two groups also did not differ significantly (P = 0.5). However, the mean serum vitamin B12 level was lower in the metformin group (*P* < 0.01), and the mean folate level was higher in the non-metformin group (*P* < 0.01). There were also significant differences between the two groups (P < 0.001) in homocysteine and vitamin D, with mean levels of both higher in the metformin group. Mean levels of FBS and HbA1c did not differ significantly between the two groups (P = 0.5 and 0.1, respectively) and there was no significant difference between the two groups in vildagliptin, pioglitazone, and sulphonylurea intake (P = 0.2, 0.9, and 0.2, respectively). Insulin use was higher in the metformin group, with an almost twofold greater frequency of insulin use compared with the non-metformin group (63% vs. 33.3%, *P* < 0.01). The average duration of diabetes was also higher among the metformin users, by approximately 2.8 years (*P* < 0.01). The majority of metformin users (196; 61.13%) had been using it for more than 4 years, while 123 (38.56%) had been using it for 1–4 years, and only 1 patient (0.31%) had been using it for less than 1 year. The vast majority of metformin users (301; 94.36%) were on a regimen of 1000–2000 mg/day, while 12 (3.76%) and 6 (1.88%) were on regimens of >2,000 mg/day and ˂1000 mg/day, respectively. None of the patients in the non-metformin group used H2 blockers. Out of the vast majority of patients administered metformin (315; 98.75%), only 2 (0.63%) had been taking H2 blockers for less than 1 year, and the same number (2, 0.63%) had been taking H2 blockers for more than 1 year (P = 0.9). Proton pump inhibitor (PPI) intake was higher among the metformin users (*P* < 0.01). Among those users taking a PPI for less than 1 year, the percentage of metformin users was higher than the percentage of non-metformin users (19.1% vs. 10.8%). Metformin users were also overrepresented among patients taking a PPI for more than 1 year (16% vs. 1.1%). The prevalence of hypothyroidism did not differ significantly (P = 0.1) among the two groups. Heart failure and other comorbidities were higher in the non-metformin users (*P* < 0.05 and *P* < 0.01 for heart failure and other comorbidities, respectively). However, no significant differences were found between the two groups in stroke (P = 0.2) or ischemic heart disease (P = 0.1). The overall prevalence of vitamin B12 deficiency in all participants with T2DM was 7.8% (32 out of 412). Compared with non-metformin users, metformin users had a significantly higher prevalence of vitamin B12 deficiency (9.4% vs. 2.2%, *P* < 0.036) (see Table 2).

**Table 2:** Comparison of proportion of patients suffering from vitamin-B12 deficiency between metformin and non-metformin users (N = 412).

Variables	All participants (N = 412) n (%)	Non-metformin user (n = 93) n (%)	Metformin user (n = 319) n (%)	P-value
**Normal serum vitamin B12**	380 (92.23%)	91 (97.8%)	289 (90.6%)	< 0.036
Deficient serum vitamin B12*	32 (7.77%)	2 (2.2%)	30 (9.40%)	

*The cut-off for vitamin B12 deficiency was 132.8 pmol/L

The odds ratio for serum vitamin B12 deficiency in metformin users was 4.72 (95% CI, 1.11–20.15, *P* = 0.036). Table 1 shows the overall results of the analyses for neuropathy status using TCSS and focused neurological testing. No significant differences between metformin and non-metformin users were seen (*P* > 0.05 for all measures).

**Fig 1:**
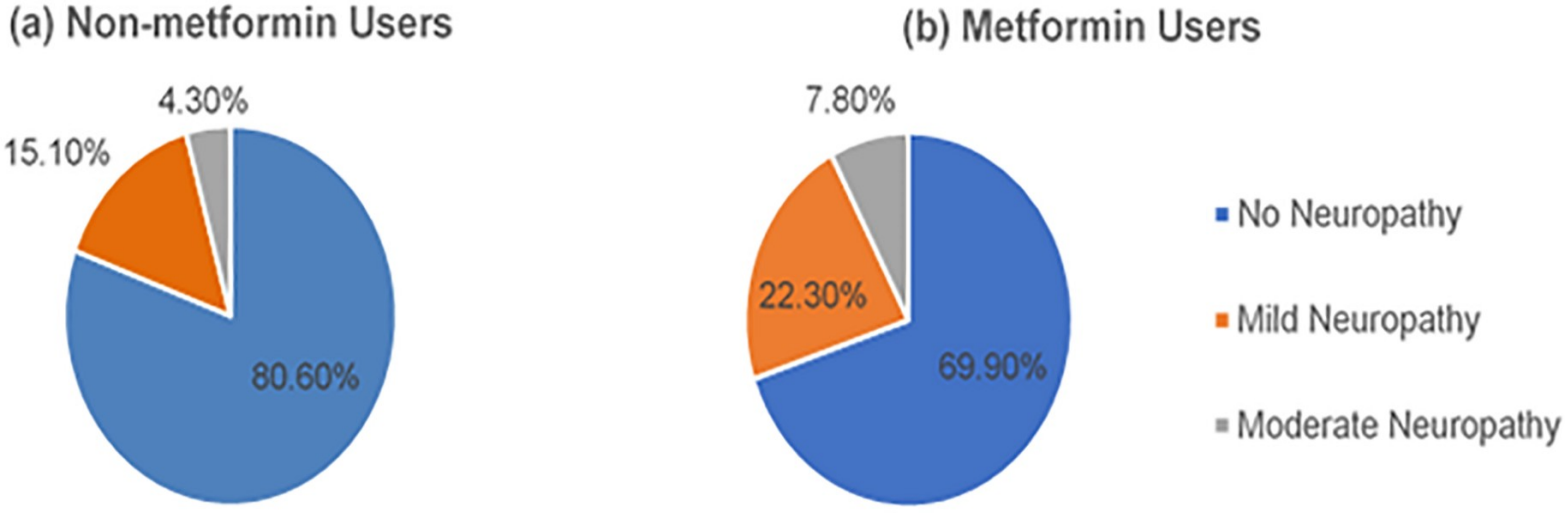
Prevalence of neuropathy in the metformin and non-metformin groups, based on the Toronto Clinical Scoring System.

Fig 1 shows that the metformin group had higher frequencies of mild and moderate neuropathy than the non-metformin group (mild status, 22.3% vs. 15.1%; moderate status, 7.8% vs. 4.3%, respectively).

**Fig 2:**
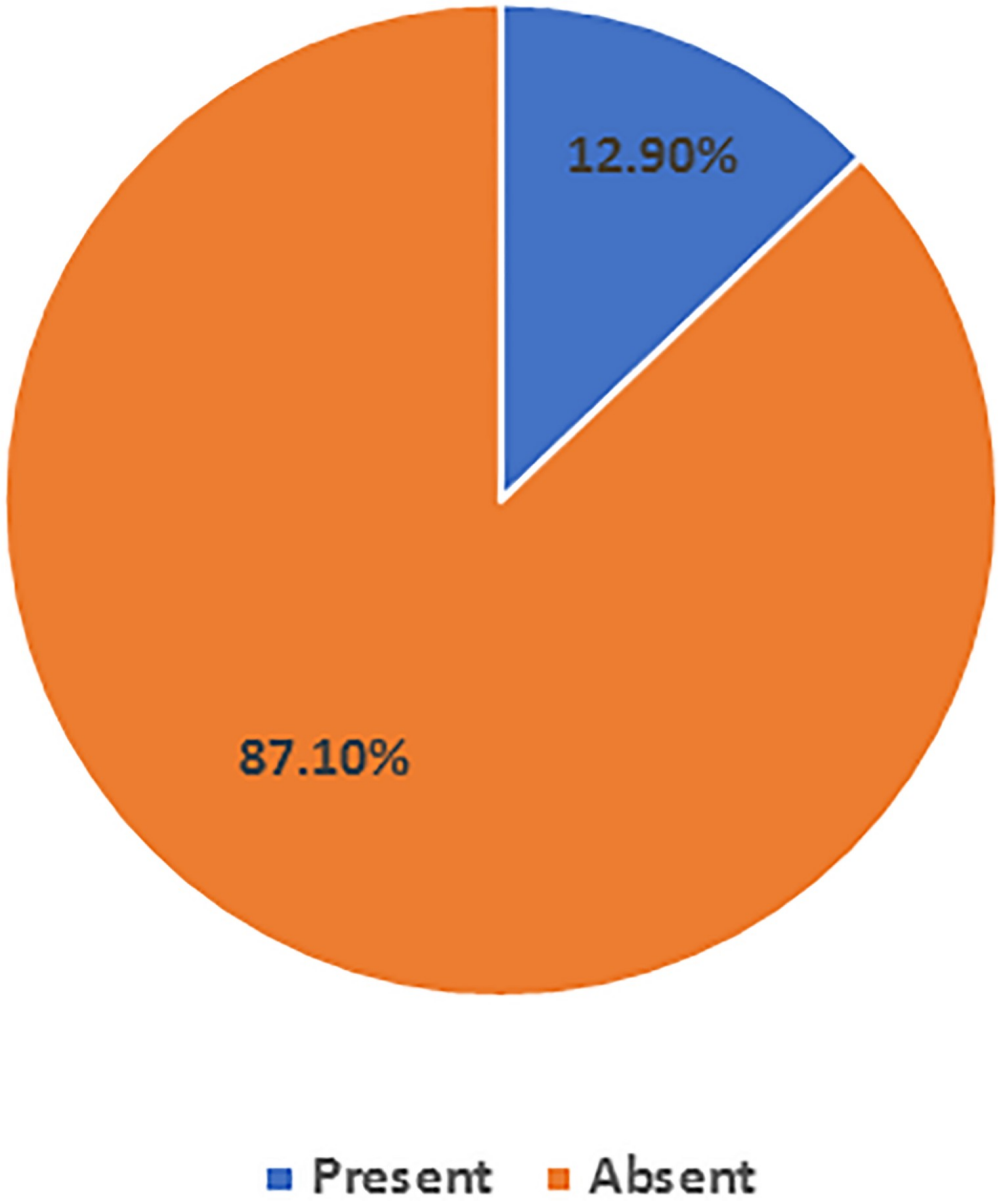
Prevalence of neuropathy, as assessed by the focused neurological test (N = 412).

Fig 2 illustrates the result for the focused neurological examination. The clinical characteristics for the group with serum vitamin B12 deficiency and the group with normal serum vitamin B12 are shown in Table 3.

**Table 3:** Clinical characteristics of the participants, stratified by B12 deficiency status (N = 412).

Characteristic	Vitamin B12 deficiency		OR (95% CI)*	*P*-value†
	Yes (n = 32) Mean (SD) or n (%)	No (n = 380) Mean (SD) or n (%)		
**Homocysteine (μmol/L)**‡	31.2 ± 2.1	19.3 ± 1.0		
**Normal**	2 (6.25)	256 (67.37)	Ref.	<0.01
**High**	30 (93.75)	124 (32.63)	30.97 (7.28, 131.67)	
**Folate (nmol/L)**§	20.7 ± 1.8	24.2 ± 0.5		
**Normal**	26 (81.25)	341 (89.74)	Ref.	
**Low**	4 (12.5)	30 (7.89)	1.75 (0.57, 5.34)	0.49
**High**	2 (6.25)	9 (2.37)	2.91 (0.60, 14.20)	0.19
**B12 intake (mcg/week)**	17.6 ± 0.77	24.1 ± 0.45		
**Below RMDI‖**	12 (37.5)	80 (21.05)	Ref.	
**Above or equal RMDI**	20 (62.5)	300 (78.95)	0.44 (0.21, 0.95)	0.045
**BMI**¶	35.1 ± 1.25	32.2 ± 0.45		0.060
**Metformin Dose (mg)**				
**0**	2 (6.25)	91 (23.95)	Ref.	
**< 1,000**	0 (0)	6 (1.58)	3.79 (0.15, 93.70)	0.20
**1,000–2,000**	25 (78.13)	276 (72.63)	4.12 (0.96, 17.74)	0.05
**> 2,000**	5 (15.62)	7 (1.84)	32.5 (5.31, 198.81)	<0.01
**Duration of Metformin Intake (years)**				
**0**	2 (6.25)	91 (23.95)	Ref.	
**< 4**	6 (18.75)	117 (30.79)	2.33 (0.46, 11.83)	0.47
**> 4**	24 (75)	172 (45.26)	6.35 (1.47, 24.47)	<0.01
**Use of PPI**◊				
**0**	11 (34.38)	278 (73.16)	Ref.	
**< 1**	8 (25)	63 (16.58)	3.21 (1.24, 8.31)	0.02
**> 1**	13 (40.62)	39 (10.26)	8.42 (3.53, 20.11)	<0.01
**Toronto Clinical Scoring**				
**No Neuropathy**	25 (78.13)	273 (71.84)	Ref.	
**Mild**	7 (21.87)	78 (20.53)	0.98 (0.41, 2.35)	1.00
**Moderate**	0 (0)	29 (7.63)	0.19 (0.01, 3.17)	0.28
**Focused Neurological test**				0.78
**Negative**	29 (90.63)	330 (86.84)	Ref.	
**Positive**	3 (9.37)	50 (13.16)	1.46 (0.43, 4.99)	0.60

* OR: Odds Ratio

^†^ Fisher’s exact test and the Chi-squared test of association are used for the comparison between categorical variables, and two-samples independent t-test is used for the comparison between continuous variables.

^‡^ Homocysteine normal level 4–15 μmol/L

^§^ Folate normal level: 10.4–42.4 nmol/L

^‖^ RMDI: Recommended Minimum Daily Intake (2.4 mcg/day or 16.8 mcg/week)

^¶^ BMI: Body Mass Index

^◊^ PPI: Proton-pump inhibitor

There was no significant difference between the mean BMI values of the two groups (P = 0.06), nor were there significant differences in TCSS and focused neuropathy test results, with TCSS values of 0.98 (0.41, 2.35) and 0.19 (0.01, 3.17) and focused neurological test results of 1.46 (0.43, 4.99). The individuals with vitamin B12 deficiency had significantly (P ˂ 0.01) higher mean serum homocysteine levels [30.968 (7.82, 131.667)] and lower mean folate levels (20.7 ± 1.8 vs. 24.2 ± 0.5 nmol/L; *P* < 0.01) with an OR of 1.75 (0.57, 5.34) and 2.91(0.60, 12.20). The group with vitamin B12 deficiency had been taking a significantly higher daily dose of metformin (*P* < 0.01), and had been taking it for longer (*P* < 0.01), than the groups without vitamin B12 deficiency, with ORs for metformin dose of 0.01 (0.01, 0.88), 2.14(0.96–17.74), and 32.5 (5.31, 198.8), and ORs for metformin duration of 2.33 (0.46, 11.83) and 6.35 (1.47, 24.47). Also, the group with vitamin B12 deficiency had higher PPI intake (*P* < 0.01), OR: 3.21 (1.24, 8.31) and 8.42 (3.53, 20.11), and lower weekly vitamin B12 consumption (*P* < 0.01) with OR for B12 intake of 0.44 (0.21, 0.95).

**Fig 3:**
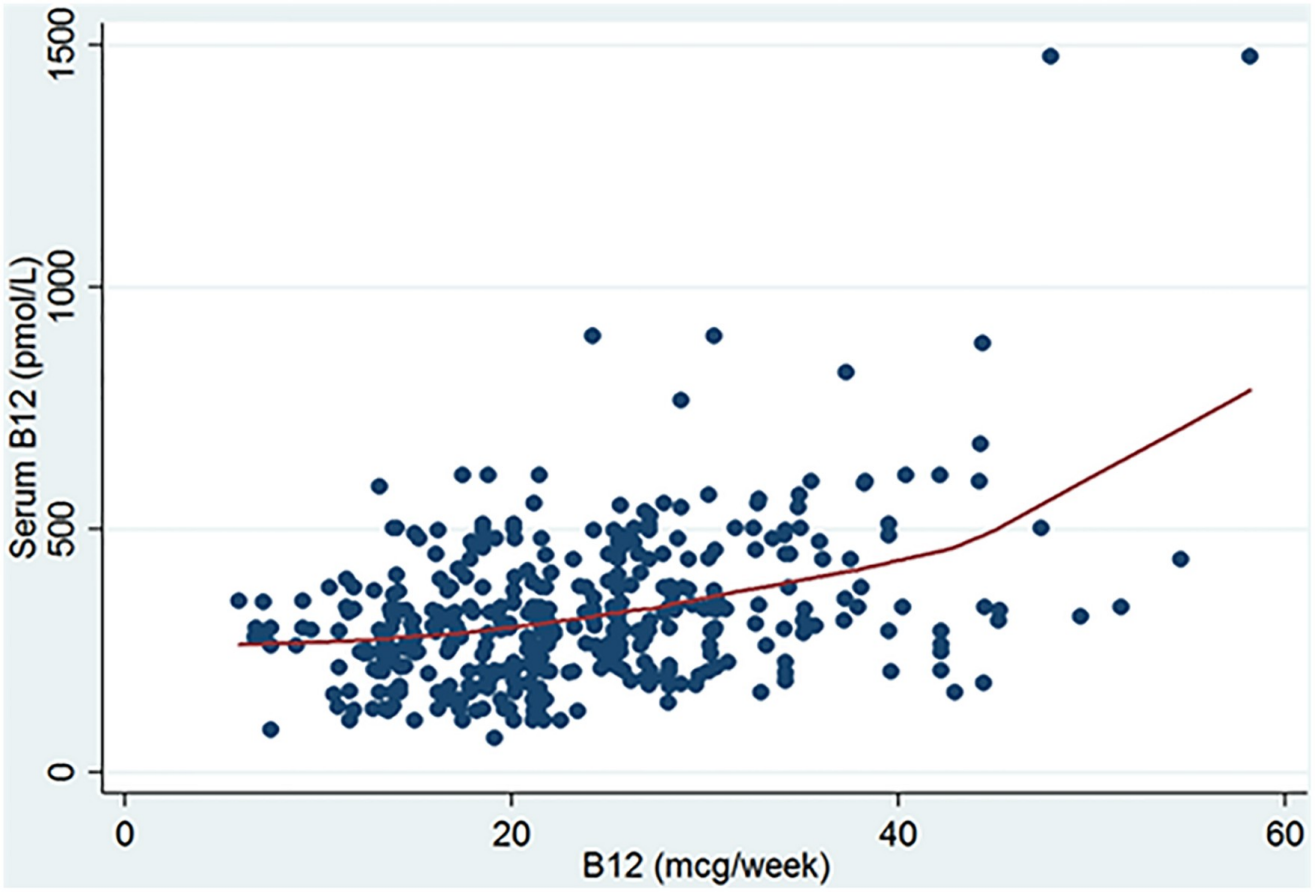
Non-parametric LOWESS curve with a scatter plot for the relationship between the average weekly vitamin B12 consumption and serum vitamin B12 (N = 412).

Fig 3 shows a positive correlation between the weekly consumption of vitamin B12 and serum vitamin B12. Fitted, nonparametric locally weighted scatterplot smoothing (LOWESS) suggested a nonlinear positive correlation. This correlation may have confounded the results of our analysis if we had not taken it into consideration. Moreover, the Pearson correlation coefficient results suggest a moderate overall correlation between weekly consumption of vitamin B12 and serum level of vitamin B12 (r = 0.39, *P* ˂0.001). However, the stratification based on metformin use shows that such a correlation was slightly decreased in the metformin group (r = 0.35, *P* ˂0.001), and was increased in the non-metformin group (r = 0.55, *P* ˂0.001) (see Table 1A).

The statistical modeling of the relationship between B12 deficiency and demographics and clinical characteristics is presented in Table 4.

**Table 4:** Unadjusted and adjusted logistic regression using the presence of B12 deficiency as a dependent variable with other clinical characteristics as independent variables (N = 412).

Variable	Unadjusted OR (95% CI)*	P-value	Adjusted OR (95% CI)**	*P*-value
**Gender**				
**Male**	Reference	Ref.	Reference	<0.01
**Female**	7.15 (2.46, 20.77)	<0.01	14.08 (4.09, 48.45)	
**Age**	0.9 (0.9, 1.0)	0.19	Not included†	
**B12 intake (mcg/week)**	0.89 (0.84, 0.94)	<0.01	0.82 (0.76, 0.89)	<0.01
**BMI**	1.03 (1.00, 1.07)	0.08	Not included†	
**Duration of Diabetes (years)**	1.01 (0.97, 1.06)	0.63	Not included†	
**Duration of Metformin Intake (years)**			Not included†	
**0**	Reference	Ref.		
**< 4**	2.33 (0.46, 11.83)	0.31		
**> 4**	6.35 (1.47, 24.47)	0.01		
**Metformin Dose (mg)**				
**0**	Reference	Ref.	Reference	Ref.
**< 1,000**	1.00 (0.90, 1.12)	0.99	1.05 (0.15, 13.85)	0.71
**1,000–2,000**	4.12 (0.96, 17.74)	0.06	3.81 (0.92, 14.92)	0.06
**> 2,000**	32.50 (5.31, 198.80)	<0.01	21.67 (2.87, 163.47)	<0.01
**Use of PPI**			Not included†	
**0**	Reference	Ref.		
**< 1**	3.21 (1.24, 8.31)	0.02		
**> 1**	8.42 (3.53, 20.11)	<0.01		
**Use of H2 Blocker (years)**			Not included†	
**0**	Reference	Ref.		
**< 1**	12.16 (0.74, 199.17)	0.08		
**> 1**	1.61 (0.63, 18.42)	0.28		
**Hypothyroidism**			Not included†	
**Absent**	Reference	Ref.		
**Present**	0.97 (0.41, 2.32)	0.95		
**HbA**_**1c**_	0.69 (0.52, 0.92)	0.01	Not included†	
**MCV**	0.98 (0.97, 0.99)	<0.01	0.98 (0.96, 0.99)	<0.01
**Hgb**	0.83 (0.74, 0.93)	<0.01	Not included†	

* OR: Odds ratio, CI: Confidence Interval

** The model is adjusted for metformin intake, gender, age, income, B12 weekly intake, BMI, Duration of diabetes, Duration of metformin intake. Metformin dosage, PPI, H2 Blocker, hypothyroidism, HbA1c, MCV, Hgb.

^†^ The final model selection included variables that produced the model with the lowest AIC and BIC. The rest of the variables are omitted. The final model has AIC = 143.57 and BIC = 175.52

Several variables showed significant risk or a protective effect in the unadjusted form. When the relationship was adjusted to include several covariates, however, the results showed a dramatic change in many variables. The final adjusted model includes gender, B12 weekly intake, metformin dose, and MCV. Females are at higher risk for vitamin B12 deficiency (AOR, 14.08; 95% CI, 4.09–48.5, *P* < 0.01). Each 1 mcg increase in the weekly consumption of vitamin B12 decreases the risk of B12 deficiency by 18% (AOR, 0.82; 95% CI, 0.76–0.89, *P* < 0.01). Metformin dosage in excess of 2,000 mg increases the risk of vitamin B12 deficiency by approximately 22 folds (AOR, 21.67; 95% CI, 2.87–163.47, *P* < 0.01). Each 1-unit increase in the MCV decreased the risk of B12 deficiency by 2% in the adjusted model (AOR, 0.98; 95% CI, 0.96–0.99, *P* < 0.01). The model with the lowest information criterion showed a high predictive level, with an AUC equal to 0.91 (95% CI, 0.86–0.96).

## Discussion

In our study, the overall prevalence of vitamin B12 deficiency was 7.8% (32 out of 412). Vitamin B12 deficiency levels differed in the two groups, however, with a level of 9.4% (30 out of 319) in the metformin group and 2.2% (2 out of 93) in the non-metformin group. The percentage of abnormally high levels of serum homocysteine was greater in the vitamin B12-deficient group than in the group with normal vitamin B12 levels (93.8% vs. 32.6% respectively, *P* < 0.01).

Low levels of serum vitamin B12 occurred when metformin was taken in a dose greater than 2,000 mg/day and for a period exceeding 4 years. Our observation of a correlation of vitamin B12 deficiency with metformin use is consistent with the results of previous studies [12–16]. In our study, the prevalence of vitamin B12 deficiency in metformin users was quite similar to the results reported by Ko S-h et al. (2014) [17]. Also, Chapman, in a systematic review, indicated that the findings of 17 cross-sectional studies showed that there is an association between lower levels of vitamin B12 and metformin use [8]. However, other studies provided contrary results; these studies showed that metformin might improve metabolism of vitamin B12 and only lowered the inactive form of vitamin B12 (holo-haptocorrin, holoHC) rather than the active form of vitamin B12 (Holotranscobalamin, holoTC) [5, 18–23]. Long-term metformin use reduced vitamin B12 levels and worsened neuropathy [7, 12, 24, 25]. Our present findings showed that long-term metformin use and cumulative daily metformin dosage were the most consistent risk factors associated with a significant decrease in serum vitamin B12. The mean dose (2,000 mg/day) and duration of metformin use (4 years) were higher in our study compared with previous studies [17, 26]. Therefore, comparing the findings of our study with those of earlier studies is not straightforward, and should take into consideration several factors. One of these factors is the variation in duration and dose of metformin use; duration of metformin use ranged from 16 weeks to more than 4 years, and metformin dose ranged from 1,000 mg/day to more than 2,000 mg/day [5, 14, 27–29]. Regarding the peripheral neuropathy, our finding of a high proportion of mild neuropathy in metformin users was similar to a result reported by Kamphuis et al. [30]. However, it was not in concordance with other studies, which reported a higher proportion of severe neuropathy [7, 11]. None of these studies showed any association between neuropathy and the duration of metformin therapy, which were positively associated in Gupta et al., 2018 [31].

Our study demonstrated that individuals with vitamin B12 deficiencies had a significantly higher level of serum homocysteine than individuals with normal serum vitamin B12. The majority of cases of vitamin B12 deficiency were in metformin users (30 out of 32), which indicates that metformin might influence not only vitamin B12 but also could affect serum levels of homocysteine. A similar finding was reported in other studies [28]. However, one study reported that metformin treatment has no effect, or only a small effect, on the concentration of homocysteine [9].

MCV seems to be confounded by smoking status of the participant. We therefore excluded this variable. Smoking status was a problematic variable to be included for several reasons. First, due to social norms, females underreport their smoking habits. Secondly, some participants who occasionally smoke cigarettes, waterpipes, or e-cigarettes self-report as non-smokers. Therefore, we opted for excluding this variable due to the potential for bias. Moreover, the relationship between MCV and vitamin B12 deficiency varied among studies. Nishant Raizada et al. (2017), in an examination of the impact of vitamin B12 deficiency on hematological parameters such as MCV and hemoglobin, as well as on peripheral neuropathy, did not find any increase in MCV or decrease in hemoglobin in Vitamin B12-deficient patients.[32] Another study, D.M. de Groot-Kamphuis et al., 2013, reported anemia in 23.3% of patients using metformin, compared with 22.3% in patients not using metformin (95% CI of the difference -1.1 to 8.6; p = 0.82) [33]. In both the metformin and non-metformin groups, three patients had microcytic anemia. Mean MCV in the metformin group was 89.1 fl compared with 90.2 in the non-metformin group (p = 0.08). However, in D.M. de Groot-Kamphuis et al. 2013, the prevalence of B12 deficiency was significantly higher in patients treated with metformin compared with non-metformin users. Metformin use, however, does not predict the odds for anemia or neuropathy after 4.9 years of treatment with metformin [33].

Finally, our study demonstrated that higher dietary intake of vitamin B12 was positively associated with higher levels of serum vitamin B12. However, increased dietary intake of vitamin B12 did not protect metformin users from serum vitamin B12 deficiency. This finding was in concordance with that of Monique et al., 2010 [23].

### Strengths and limitations

The strengths of our study lie in enrollment of participants from all age groups above 18 years, with detailed information concerning metformin use and confounding factors for vitamin B12 deficiency (e.g., assessment for dietary B12 intake). This study compared the prevalence of vitamin B12 deficiency in metformin users and non-users, and subdivided metformin use on the basis of two factors: length of use and dosage per day. Moreover, neuropathy screening and neurological examination were conducted in both groups (including symptomatic and asymptomatic participants). An additional strength in this study was our measurement of homocysteine side by side with vitamin B12, which allowed homocysteine to act as an additional biomarker to improve the sensitivity of detection of vitamin B12 deficiency.

Our study has several limitations. The design of this study precludes the causal association between metformin use and vitamin B12 deficiency. Compliance with metformin treatment was not assessed during the study, and this may have had an impact on the change in serum vitamin B12 in response to metformin. The effect of smoking was not examined in this study but has been addressed in other studies, for example, Kham et al. [34], who found lower B12 levels in smokers than in non-smokers. Also, the patients’ intake of supplemental calcium and vitamin D, which have been shown to play major roles in vitamin B12 synthesis, was not evaluated. Assessment of megaloblastic anemia was not carried out; therefore, the clinical implications of this deficiency were not followed, and the effect of replacement of vitamin B12 was not assessed. Electrodiagnostic tests for neuropathy were not conducted for our participants. Instead, our participants were screened for neuropathy using validated scales. Additionally, this study did not assess all confounders of neuropathy. Finally, metformin users had a higher intake of PPI than non-metformin users, which could lead to vitamin B12 deficiency; however, our study adjusted for this via logistic regression analysis.

## Conclusion

In conclusion, in our study, the prevalence of vitamin B12 deficiency was higher in metformin users than in non-metformin users. There was also an association between vitamin B12 deficiency and the dose and duration of metformin use. Therefore, we recommend routine screening for serum vitamin B12 in individuals with T2DM who take daily metformin doses higher than 2,000 mg, or for a duration exceeding 4 years.

## Supporting information

S1 Table Correlation coefficient between B12 weekly consumption and level of serum B12 (N = 412). (PDF)

S1 File VitB12data. (DTA)
