# Supplementary material for: The association of metformin use with vitamin B12 deficiency and peripheral neuropathy in Saudi individuals with type 2 diabetes mellitus
Source: PLoS One. 2018 Oct 15;13(10):e0204420. doi: 10.1371/journal.pone.0204420 (PMC6188756; doi:10.1371/journal.pone.0204420)
Supplement: S1 Table — (PDF) [file pone.0204420.s001.pdf]

Appendix

Table 1A: Correlation coefficient between B12 weekly consumption and level of serum B12 (N = 412).

	**B12 consumption (mcg/week)**		
	**Group**		
	**All (N = 412) r (*P*-value)**	**Non-metformin user (n = 93) r (*P*-value)**	**Metformin user (n = 319) r (*P*-value)**
Serum vitamin B12	0.384 (*P* = <0.001)	0.55 (*P* = <0.001)	0.35 (*P* =<0.001)
